# Beneficial Effects of Green Tea EGCG on Skin Wound Healing: A Comprehensive Review

**DOI:** 10.3390/molecules26206123

**Published:** 2021-10-11

**Authors:** Fa-Wei Xu, Ying-Li Lv, Yu-Fan Zhong, Ya-Nan Xue, Yong Wang, Li-Yun Zhang, Xian Hu, Wei-Qiang Tan

**Affiliations:** 1 Department of Plastic Surgery, Sir Run Run Shaw Hospital, Zhejiang University School of Medicine, 3 East Qingchun Road, Hangzhou 310016, China; xufawei@zju.edu.cn (F.-W.X.); 12018261@zju.edu.cn (Y.-F.Z.); xueyanan2020@zju.edu.cn (Y.-N.X.); wongyong@zju.edu.cn (Y.W.); 18868735326@163.com (L.-Y.Z.); huxiandxg@163.com (X.H.); 2 Tea Research Institute, College of Agriculture and Biotechnology, Zhejiang University, Hangzhou 310013, China; 3180100543@zju.edu.cn

**Keywords:** EGCG, skin wound healing, wound dressing, anti-inflammation, angiogenesis, antifibrosis

## Abstract

Epigallocatechin gallate (EGCG) is associated with various health benefits. In this review, we searched current work about the effects of EGCG and its wound dressings on skin for wound healing. Hydrogels, nanoparticles, micro/nanofiber networks and microneedles are the major types of EGCG-containing wound dressings. The beneficial effects of EGCG and its wound dressings at different stages of skin wound healing (hemostasis, inflammation, proliferation and tissue remodeling) were summarized based on the underlying mechanisms of antioxidant, anti-inflammatory, antimicrobial, angiogenesis and antifibrotic properties. This review expatiates on the rationale of using EGCG to promote skin wound healing and prevent scar formation, which provides a future clinical application direction of EGCG.

## 1. Introduction

The skin is the first line of defense against external aggressors. The skin’s integrity can be damaged by trauma, tears, cuts or contusions, resulting in skin wounds. Full-thickness wounds that extend beyond the two layers of skin (dermis and epidermis) heal through a granulation process and scar formation [1]. Scars are apparently distinguished from the surrounding skin (e.g., darker colour, stretched, depressed or raised), and may also have various symptoms such as inflammation, erythema, pruritus and pain. In some cases, pathological scars (e.g., hypertrophic and keloid scars) inevitably form, adversely impacting sufferers’ life quality. In addition, some diseases such as diabetes impede wound healing process through causing long-term inflammation [2]. Wound healing requires suitable environment to promote healing process, e.g., optimal moisture and redox environment [3,4]. Plant polyphenols as natural antioxidant agents are cost-effective alternatives to current pharmacologic therapeutics, which have been formulated or fabricated in wound dressings to improve the conditions for wound healing [5,6].

Green tea is characterized by the high content of polyphenols, which is produced from the tea plant *Camellia sinensis*. (−)-Epigallocatechin gallate (EGCG) is regarded as the most abundant compound in tea leaves with excellent bioactivities, such as antioxidant/free radical scavenging [7], anti-inflammatory and antimicrobial properties [8,9]. However, the clinical application of EGCG is restricted by its low bioavailability, since EGCG is unstable under the alkalescent condition of the intestinal track and circulatory system [10]. It was reported that a single injection of EGCG hardly accelerated the healing process of the wound on the back of rats [11]. Thus, topical application might be an ideal route to fully achieve the functionalities of EGCG, considering the avoidance of gastrointestinal digestion and less adverse effects on other organs. The potential application of EGCG to skin wound treatment has been investigated, and some positive results in vitro and in vivo have been achieved [12,13,14,15]. The effects of EGCG on wound healing are associated with the application form and the dosage of EGCG, study models and treatment methods.

This review appraises the developments of EGCG-containing wound dressings for skin wounds. The actions of EGCG in different healing processes are elaborated, and the underlying mechanisms are summarized, including anti-inflammatory, antioxidant, antimicrobial, angiogenesis and antifibrotic effects. This review provides a reference for developing EGCG as a wound-healing agent for clinical use.

## 2. The Application Forms of EGCG to Wound Healing

**Figure 1:**
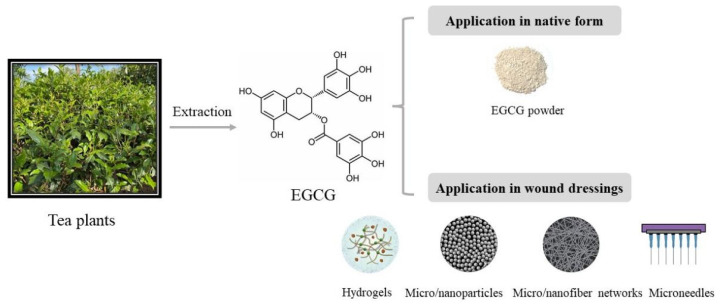
The native and wound dressing forms of EGCG.

Since many phytonutrients, such as EGCG, are water soluble, neither aqueous solutions nor powders are feasible for clinical applications due to their low adhesion abilities and the limited permeabilities of the skin. Hence, various types of wound dressings have been developed for topical treatments [16,17]. Except for the native form of EGCG used in many studies [13,18], EGCG can also be incorporated into different types of wound dressings, such as hydrogels [19,20], nanoparticles [21,22] and electrospun fibers [23], in order to retain its biofunctionalities and integrate it with the surrounding host tissues [24]. Figure 1 shows the basic application forms of EGCG to skin wound healing. Biomaterials used for cutaneous delivery systems have to meet several requirements, such as biocompatibility, biodegradation, good permeability to moisture and oxygen, good adhesive and infiltrative properties, as well as a reduction in infections and mechanical irritations [25].

Hydrogels are insoluble hydrophilic materials with high water content (70–90%) and a soft elastic property [26] which can be produced from synthetic polymers or natural macromolecule polymers. High-molecular polymers, e.g., poly (methacrylates) and polyvinyl pyrrolidine, are artificial polymers that are widely used, while polysaccharides (e.g., chitosan, hyaluronic acid, alginate and cellulose) and proteins (e.g., collagen, alginate and gelatin) are common natural hydrogels [12]. Hydrogels have been used for translational medicine and sealants with the advantages of better tissue adhesive character [19,27], rapid gelation and injectable properties [28,29] as well as good biocompatibility [30]. EGCG was conjugated with hyaluronic acids, and the conjugates further reacted with tyramine-conjugated hyaluronic acids through tyrosinase to form a crosslinked adhesive hydrogel [20]. A simplified one-pot synthesis method of EGCG–chitosan hydrogels were developed through enzyme-mediated cross-linking [12]. EGCG was copolymerized with 3-acrylamido phenyl boronic acid and acrylamide to form a hydrogel with adequate mechanical properties and tissue adhesiveness [31]. In addition to direct complexation with bioactives, hydrogels can also be used as a carrier of fabricated structures to improve their physical properties, such as nanoparticles.

Microencapsulation is a protective technology of encapsulating bioactives into micro/nanoparticles, with the range of 10 to 100 nm for nanoparticles and a larger range up to 800 μm for microparticles [10,32]. Microencapsulation technology has been widely used in the fields of medicine, functional foods and cosmetics [10,33,34]. Metal nanoparticles, particularly gold, silver and copper nanoparticles or bimetallic nanostructures, showed the excellent potential for wound treatment due to their unique surface and electronic properties. However, the toxicity of metal nanoparticles is still a concern to the public. Nanoparticles are usually carried by other matrices in the forms of ointment, gel or a patch to achieve tissue adhesive, good compatibility and anti-inflammatory properties while reducing cytotoxicity [22,35]. Natural biomacromolecules, such as chitosan and gelatin, are also commonly used materials for constructing microparticles [10]. EGCG–gelatin–chitosan microparticles were constructed and incorporated into a poly(γ-glutamic acid)/gelatin hydrogel to fabricate a sandwiched dressing for enhancing wound regeneration [36]. Nano- and microscale particles fabricated by synthetic polymers such as poly(lactide-co-glycolic acid) (PLGA) were also used for incorporating EGCG to promote wound healing, with the advantages of biocompatibility and a sustainable anti-inflammatory effect [37]. In addition, some growth factors are good candidates for coating in order to endow the recombinant nanoparticles with targeted therapeutic effect in the wound area, for instance, epidermal growth factor (EGF) [38,39].

Networks composed of nano-/micrometer electrospun fibers have been developed for temporary wound dressing, which have the features of porosity, tunable wettability, degradation and biocompatibility [23,40]. Electrospinning technology is used to entrap phenolic compounds within polymer matrices [41], for example, PLGA [23,42], polycaprolactone (PCL)/gelatin nanofibers mats [43] and polysaccharide-based nanofibrous materials [44]. The EGCG-loaded polymer matrices are associated with a sustainable release of EGCG [42], prolonged oxidation [43] and an effect of promoting wound healing [23].

Microneedles have been used for delivering bioactives via the transdermal route [45,46,47]. Microneedle devices can help the bioactives pass through the epidermis, led by these micro-needles [48]. Biomaterials, such as chitosan [49], hyaluronic acid [45] and maltose [46], are used to fabricate microneedles through in situ polymerization using a mold-based technique. Microneedles loaded with green tea extract were prepared and showed the potential therapeutic effect on wound healing based on the antibacterial property [45]. The peptides delivered by poly(ethylene glycol) diacrylate microneedles inhibited the expression of collagen I to suppress the formation of keloid scars [50]. Although there is still no report about the direct application of EGCG-loaded microneedles to skin wound treatment, the microneedle-mediated intradermal delivery system has been used to lead EGCG to deeper skin layers for dermal applications [46], e.g., atopic dermatitis treatment [51]. This implies the great potential of using EGCG-loaded microneedle devices to promote skin wound healing. No matter what topical application forms of EGCG are used, the promoting effects of EGCG on wound healing are based on its basic pharmacological functions.

## 3. Beneficial Effects of EGCG at Different Healing Stages

The skin wound healing process has four sequential and overlapping stages, including hemostasis, inflammation, proliferation and tissue remodeling [52], which involves various types of cells (e.g., leukocytes, fibroblasts and keratinocytes) and several factors, such as cytokines, chemokines, growth factors and enzymes. These cells and factors are differentially featured at each wound healing stage [53].

Hemostasis happens very quickly after injury, which is accompanied by clotting. As injury occurs, platelets stick together to seal the break in vessel, followed by coagulation and the formation of a platelet plug. In addition, platelet activation also leads to the activation of the immune system and the transition to the inflammatory phase through the release of cytokines and growth factors, such as transforming growth factor β (TGF-β), EGF, platelet-derived growth factor (PDGF) and fibroblast growth factor (FGF) [52,54]. Hemostatic hydrogel is developed for halting bleeding quickly, and good adhesiveness, self-recovery capacity and antibacterial properties are desired [55]. A hemostatic hydrogel was prepared by adding self-assembled keratin–EGCG nanoparticles into cellulose hydrogel, which not only improved the physical properties of pure keratin materials but also exhibited good adhesiveness and hemadsorption [56]. As bleeding is controlled, the inflammatory phase starts, which is characterized by the recruitment of neutrophils, macrophages and lymphocytes.

The inflammatory phase is critical to clear out pathogenic organisms and create a suitable environment for the subsequent tissue repair and regeneration phase [52]. The inflammation phase occurs shortly after injury (first 48 h), which is characterized by the transduction of signaling cascades, the recruitment of neutrophils, monocytes and macrophages at the wound area as well as the release of various growth factors, cytokines and chemokines [53,54]. In the wound environment, neutrophils upregulate the gene expressions of chemokines, such as tumor necrosis factor α (TNFα), interleukin (IL)-1β, IL-6, IL-8, vascular endothelial growth factor (VEGF) and monocyte chemoattractant protein-1 (MCP-1); recruit macrophages, T cells and additional neutrophils; and promote angiogenesis and proliferation of fibroblasts and keratinocytes [52,57]. Monocytes arrive at the wound and differentiate into macrophages or dendritic cells. Macrophages are responsible for phagocytosing apoptotic neutrophils, removing bacteria and dead cells in the wound area and cooperating with neutrophils during the inflammatory phase [58]. Macrophages also secrete cytokines such as PDGF, TGF-β, β-FGF, TNFα, IL-1 and IL-6 [53]. In addition, macrophages phenotypically transit to a reparative state that resolves from inflammation and stimulates keratinocytes and fibroblasts for the subsequential tissue regeneration [54], promoting the transition to the proliferative phase. During the inflammatory phase, neutrophils are the main cells that produce proteases and reactive oxygen species (ROS) that cause cell damage if not properly controlled [54]. EGCG was reported to have the inhibitory effects on the infiltration of neutrophils [59] as well as the migration and adhesion of monocytes [60]. In light of the claimed anti-inflammatory property of EGCG, many studies have been carried out to investigate the inhibitory effect of EGCG alone or combined with other phytonutrients on the generation of pro-inflammatory cytokines (e.g., TNFα, IL-1β, IL-8 and IL-6) in relevant skin cells and the wound tissues of tested animals, and positive results were achieved [11,13,61].

The proliferative phase involves the re-establishment of vessels, the generation of granulation tissue and the re-epithelialization of the wound surface. At this stage, fibroblasts are the major cells involved in the formation of granulation tissue, macrophages are the dominant inflammatory cells during the proliferative phase of skin wound repair [53] and keratinocytes are the predominant cells in the epidermis for epithelialization [62]. The interactions between keratinocytes and fibroblasts are critical for skin repair [63]. The new tissue in the wound area is generated based on collagen and an extracellular matrix (ECM), both of which are mainly synthesized by fibroblasts. Several molecules derived from macrophages, such as TNFα, IL-1 and IL-6, can induce the generation of pro-re-epithelialization molecules in fibroblasts [64]. At the early stage of tissue repair, fibroblasts start to differentiate into α-smooth muscle actin (SMA)-expressing myofibroblasts that actively produce ECM proteins for wound contraction, and myofibroblasts reach a peak number in the proliferation phase [65]. Myofibroblast-induced fibrosis can be overactivated by TGF-β, IL-4 and IL-13 [66]. The long-term overactivation of fibroblasts and myofibroblasts is associated with excessive collagen production and aberrant scar formation [67], and the TGF-β/Smad signaling pathway is regarded as a canonical pathway of regulating the collagen formation in fibroblasts and myofibroblasts [67]. EGCG upregulated the gene expression of klotho in normal human epidermal keratinocytes through protein kinase A (PKA)-cAMP responsive element-binding protein (CREB) signaling, leading to the differentiation of keratinocytes [68]. EGCG mediated the TGF-β1-induced collagen contraction in fibroblasts through suppressing myofibroblast differentiation and reducing the gene expressions of connective tissue growth factor and collagen type I gene [69]. In a human keloid organ culture, EGCG reduced the generation of collagen-I and -III at the transcriptional and protein levels, depleted the mast cells and decreased the cellularity and blood vessel count after 4 weeks of treatment [70]. The animal studies showed that the applications of EGCG and their wound dressings promoted the wound healing process [36,59,71]. An EGCG/PLGA membrane treatment greatly improved the infiltration of surrounding cells and increased the immunoreactivity of Ki-67 (re-epithelialization marker) and CD 31 (blood vessel formation marker) at the wound site of nude mice, implying that EGCG/PLGA membrane treatment is beneficial to wound healing by promoting cell infiltration, re-epithelialization and angiogenesis [23]. The gelatin and chitosan nanoparticles of EGCG and ascorbic acid have promoting effects on collagen accumulation and angiogenesis but an inhibitory effect on the infiltration of inflammatory cells at the wound area of diabetic mice [72]. The in vivo study showed an EGCG-containing sandwiched dressing facilitated wound tissue regeneration and accelerated the healing process [36]. An accelerated skin regeneration was also observed in the treatment with EGCG–chitosan hydrogels [12]. EGCG exerted a blocking effect on the TGF-β1/Smad signal transduction in anaplastic thyroid carcinoma cells [73], suggesting its prospect of suppressing the formation of aberrant scars.

Remodeling refers to the transition process from granulation tissue to scar, which can last up to a year. This progress involves the clean-up of inflammatory cells, the deceleration of angiogenesis and the replacement of type III collagen in granulation tissues with type I collagen. Paralleled fibrils are formed, leading to a low cellularity scar. Myofibroblasts are responsible for carefully coordinating the breakdown of the granulation tissue and its replacement with the stronger type I collagen [52,74], which progressively vanish in the later remodeling phase. The impact of EGCG on scar maturation is still not clear. Table 1 lists the roles of EGCG and its wound dressings in the biophysiological events during the wound healing process. EGCG has various positive effects on wound healing at the stages of inflammation and proliferation.

## 4. Mechanisms Underlying the Beneficial Effects of EGCG on Skin Wound Healing

### 4.1. Antioxidant Effect

Reactive oxygen species (ROS) exert adverse effects on cells and tissues. Generally, low ROS levels are conducive to the activation of cell signaling pathways and angiogenesis, whereas high ROS levels induce oxidative stress and compromise tissue repair, leading to chronic nonhealing wounds accompanied by inflammation [76]. Abundant phytonutrients, also known as natural antioxidants/free radical scavengers, are able to protect tissues from oxidative damage. The antioxidant effect of EGCG as a bioactive component during skin wound healing has been testified in both cell and animal studies.

H_2_O_2_, UV radiation and chemical reagents, such as Rosup agent, can be used to induce the oxidative stress of skin cells [31,43,77]. In a H_2_O_2_-induced human dermal fibroblast injury, EGCG exerted antioxidant ability by enhancing the activities of superoxide dismutase (SOD) and plasma glutathione peroxidase (GSH-Px) while decreasing the malonaldehyde (MDA) level [77]. The EGCG released from polycaprolactone/gelatin nanofibers scavenged the toxic ROS species produced by the human fetal foreskin fibroblasts as exposed to either H_2_O_2_ or UV radiation and also reduced the oxidative damage to the growth of cells [43]. Zhao et al. (2021) reported that the EGCG-3-acrylamido phenyl boronic acid-acrylamide (EACPA) hydrogel treatment largely reduced the intracellular ROS in the L929 fibroblasts stimulated by Rosup [31].

In the wound tissues of animal models, the enzymes responsible for cytoprotection against oxidative stress are important parameters to evaluate the antioxidant effect of EGCG and its wound dressings in addition to ROS scavenging activity. The application of EGCG, α-lipoic acid and gold nanoparticles mixture (AuEA) to the wound area of BALB/c mice significantly elevated the protein level of SOD in the wound tissue, compared with the vehicle control group [78]. Heme oxygenase 1 (HO-1) is a cytoprotective enzyme responding to cellular stress [79], the induction of which is associated with the efficient wound closure and neovascularization [80]. EGCG significantly elevated the HO-1 protein level compared with the placebo, which showed the great potential for scar therapy applications [15].

### 4.2. Anti-Inflammatory Effect

Inflammation plays an important role in fighting pathogens and skin wound healing. Table 2 shows the anti-inflammatory effects of EGCG and its wound dressings. Different cell lines are used to establish inflammatory models, including keratinocytes [14], macrophages [20,31], endothelial cells and muscle cells, which are stimulated by lipopolysaccharides (LPS) or TNFα [13,14]. Clearly, EGCG in the native form or in wound dressings exerted inhibition on the generation of certain pro-inflammatory cytokines released to the supernatants of cells, such as TNFα, IL-1β and IL-8 [13,14,20], or downregulated the corresponding gene expressions in cells [31,37]. The anti-inflammatory effect of EGCG was also verified in the animal studies, with reduced levels of IL-1β, TNFα and IL-6 in the wound tissues [13,31]. In addition, the combinational effects of EGCG and other phytonutrients on the anti-inflammatory activity during skin wound healing were also reported [61]. The presence of EGCG in the mixture of ginkgo biloba leaves exerted cumulative downregulating effect on the secretion of IL-8 in the culture supernatants of normal human keratinocytes stimulated with TNFα [61].

The nuclear factor kappa B (NF-κB) pathway plays a crucial role in inflammation [8]. NF-κB can be activated under oxidative stress and translocated to the nucleus, inducing the transcription of the downstream genes such as TNFA, CXCL8 and iNOS. The upregulated gene expressions of TNFA, CXCL8 and iNOS lead to increased levels of TNFα, IL-8 and NO, respectively [8]. The pro-inflammatory effects of certain cytokines (e.g., TNFα and IL-1β) are associated with their abilities to stimulate NF-κB activation. EGCG reduced inflammation in acne by suppressing the NF-κB pathway [81]. The Notch signaling pathway regulates the cell-fate determination during development [82]. EGCG inhibited the LPS-induced inflammation response in mouse macrophages through targeting the Notch signaling pathway [13]. In addition to the verified NF-κB and Notch signal pathways in the skin cells or the wound tissues of animal studies, the roles of inflammation-related signal pathways in skin wound healing, such as mitogen-activated protein kinase (MAPK) and nuclear factor erythroid 2-related factor 2 (Nrf2) [8], are also worthy of investigations.

Different from pro-inflammatory cytokines, IL-4 and IL-10 are the anti-inflammatory cytokines known to suppress pro-inflammatory cytokine production [83]. Zhao et al. used an EGCG-loaded EACPA hydrogel to treat the wound area of diabetic C57BL/6 mice and found that the EGCG-loaded EACPA hydrogel treated wound tissue had higher levels of IL-4 and IL-10 than those of the undressed and Tegaderm film treated groups [31].

There are two phenotypes of macrophages: M1 macrophages (classically activated) and M2 macrophages (alternatively activated). M1 macrophages contribute to inflammation, while M2 macrophages promote collagen synthesis. In the Raw 264.7 macrophage cells simulated with LPS, Arginase-1 (ARG-1), CD163 and CD206, as a functional marker of the M2 phenotype [84,85], were transcriptionally upregulated upon EACPA hydrogel treatment [31]. CD68, as an M1 phenotype marker [86], was downregulated at the protein level in the wound tissue of diabetes mellitus mice treated with AuEA compared to the vehicle control group [59]. Moreover, EGCG or EGCG-containing wound dressing suppressed the responses of immune cells such as monocytes and macrophages in an in vivo mouse skin full defect model [12].

### 4.3. Antimicrobial Effect

**Figure 2:**
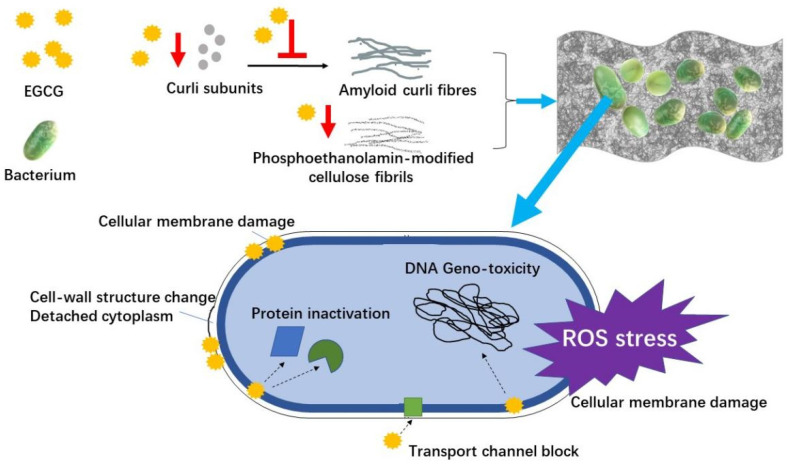
The antimicrobial mechanism of EGCG in skin wound healing process.

An infection can retard the wound healing process. Diminishing bacterial infection is an effective route to accelerate healing. *Pseudomonas aeruginosa*, *Staphylococcus aureus* and *Escherichia coli* are the common bacteria present in the wound area [22,35], which cause skin infections more frequently in the patients who have hypoimmunity [18]. Most chronic wounds in humans are involved with the formation of bacterial biofilms [87]. *Staphylococcus aureus* and *Pseudomonas aeruginosa* are able to form the biofilms that limit the penetration of antimicrobial therapeutics [35,88,89]. Figure 2 shows the antimicrobial mechanism of EGCG in the skin wound healing process, including the antimicrobial effect on bacteria and the inhibitory effect on the formation of biofilms.

Tea extract containing abundant EGCG inhibits the growth of bacteria via various ways, including disrupting cell membranes through interacting with surface proteins, decomposing essential metabolites, inhibiting relevant enzyme, inducing ROS stress, changing cell-wall structure, detaching cytoplasm, and so on [90,91,92,93]. It was reported that EGCG inhibited the glucose uptake of *Escherichia coli* through the interaction with an outer membrane porin protein, which resulted in the growth inhibition of *Escherichia coli* [94]. Thioredoxin and thioredoxin reductase are crucial to bacterial DNA synthesis and defense against oxidative stress [95]. EGCG showed an inhibitory efficacy towards thioredoxin and thioredoxin reductase in *Staphylococcus aureus* and *Escherichia coli*, leading to the suppressed growth of these pathogens [96]. The antibacterial activity of EGCG-containing gold nanoparticles (AuNPs) against *Staphylococcus aureus*, *Pseudomonas aeruginosa* and *Escherichia coli* was reported, which was attributed to the morphological deformations of bacteria due to the surface interaction with AuNPs [21]. A sandwiched dressing containing gelatin/chitosan/EGCG nanoparticles showed the antimicrobial property against *Escherichia coli*, *Pseudomonas aeruginosa* and *Staphylococcus aureus* [36]. The antibacterial activity was also verified in EGCG-containing hydrogel [12] and EGCG-containing wound patches [22].

Bacterial biofilms, mainly consisting of bacteria, polysaccharides, proteins, and lipids, fabricate a compact structure of hydrated extracellular polymeric substances [35]. EGCG interfered with the assembly of amyloid fibers from curli subunits and the generation of phosphoethanolamin-modified cellulose fibrils, which impeded the formation of biofilms [97,98]. Curli are extracellular protein fiber and functional amyloid aggregates produced by certain bacteria such as *Escherichia coli*. EGCG reduced the expression of CsgD in *Escherichia coli*, which is a key activator of curli and cellulose biosynthesis [97]. Highly fibrillation-prone protein FapC is the major component of the functional amyloid produced by many *Pseudomonas* strains. EGCG exerted an inhibitory effect on the formation of amyloids through aggregating FapC monomers into oligomers [99]. EGCG inhibited the development of biofilm formed by *Pseudomonas aeruginosa* and reduced the elastase activity, swimming and swarming motility [100]. The biofilm formed by *Staphylococcus aureus* V329 was disassembled by EGCG [101]. Due to its anti-amyloidogenic property, EGCG is regarded as an effective antimicrobial agent for preventing the formation of biofilms in chronic wound infection [98].

### 4.4. Angiogenesis Effect

Angiogenesis is the process of new branching network formation, which is mediated by various pro- and antiangiogenic factors. VEGF, as an important proangiogenic factor, can be produced by inflammatory cells [102]. The inflammatory reaction stimulated by TNFα regulates the expression of VEGF [102]. Conversely, VEGF is also involved in the regulation of inflammation, reinforcing the interrelation between inflammation and angiogenesis. The angiogenic effects of EGCG and its wound dressings are shown in Table 2. The topical treatments with EGCG-containing cream impacted the expression of VEGF, which is conducive to the prevention of telangiectasias [103]. The receptor of advanced glycation end products (RAGE) was related to oxidative stress and abnormal angiogenesis in wound healing [104,105]. The topical treatment with AuEA accelerated skin repair in diabetic mice through decreasing the transcription of RAGE and Angiopoietin-2 while increasing the gene expression of VEGF [59]. In the wound tissue of a human study, VEGFA and CD31 were reduced at both the transcriptional and protein levels under zonal priming and direct topical treatment with EGCG in first 1–2 weeks of recovery compared to the placebo control group [15]. The antiangiogenic effects of EGCG was involved in the inhibition of PI3K/AKT and MEK/ERK pathways [106].

### 4.5. Antifibrotic Effect

Fibrosis is related to abnormal repair in response to chronic tissue damage [107]. It is characterized by an increase in fibrous connective tissues in the dermis or subcutis due to the excessive proliferation of fibroblasts and the formation of collagen fibers. Fibroblasts are mesenchymal cells that play important roles in the fibrosis process. Fibroblasts are related to ECM accumulation and inflammation, contributing to fibrosis pathogenesis [108]. A keloid is a common fibroproliferative disorder related with an abnormal wound healing process [70]. Abnormal collagen synthesis leads to an imbalance in the metabolism of ECM [109]. EGCG greatly inhibited the production of type I collagen in the fibroblasts co-cultured with mast cells [109]. The antifibrotic effect of EGCG was also investigated using the model of human-derived keloid fibroblasts transplanted onto nude mice, and the productions of collagen and keloids were reduced under EGCG treatment. EGCG suppresses the pathological characteristics of keloids through inhibiting the STAT3 signaling pathway [110]. Syed et al. (2013) developed a keloid organ culture model for evaluating the antifibrotic bioactives in scarring and found that EGCG treatment decreased the size of the keloid, suppressed intrakeloid proliferation, reduced collagen production and downregulated the transcription of major fibrosis-related pathways, including VEGF, matrix metalloproteinases (MMP-2 and -9) and TGF-β2 [70]. The PI3K/AKT signaling pathway and the TGF-β signaling pathway play important roles in fibrosis [111,112], however, no relevant regulatory effect of EGCG has been reported yet.

## 5. Beneficial Effects of EGCG Derivatives on Skin Wound Healing

In addition to microencapsulation, the derivatization of EGCG is an important way to alter the physicochemical properties of EGCG, for example, methylation, alkylation and glycosylation. Epigallocatechin-3-(3″-*O*-methyl), the methylated derivatives of EGCG, scavenged the free radicals and elevated the expression of HO-1 in the keratinocytes as exposed to H_2_O_2_ [113]. Together with antibiotics, the lipid-soluble EGCG-stearate synergistically prevented the formation of biofilms produced by *Escherichia coli*, *Pseudomonas aeruginosa*, *Staphylococcus aureus*, *Staphylococcus epidermidis* and *Mycobacterium smegmatis* [114]. The alkylation of EGCG with long alkyl chains elevated its antimicrobial effect, particularly against *Staphylococcus aureus* [115]. The lipophilic derivatives of EGCG were prepared through the reaction with stearic acid, eicosapentaenoic acid and docosahexaenoic acid, which had a greater 1,1-diphenyl-2-picrylhydrazyl (DPPH) radical scavenging ability compared to EGCG [116]. Two EGCG glycosides were prepared to improve the water stability [117]. Considering the improved stability and enhanced bioactivities after derivatization reactions, the derivatives of EGCG could be used as lipophilic antioxidant or antibacterial agents for clinical usage. This provides a supplementary way of applying EGCG to skin wound repair.

## 6. Conclusions and Perspective

**Figure 3:**
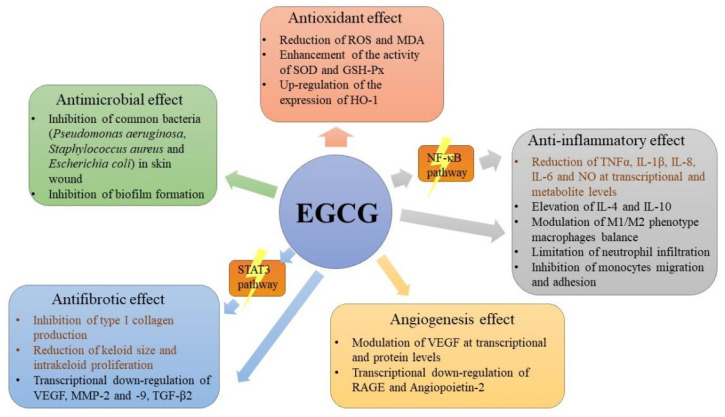
The possible mechanisms underlying the beneficial effects of EGCG on skin wound healing.

Tea has been known for its various health benefits, such as antioxidant, anti-inflammatory and antimicrobial effects due to the high amounts of catechin compounds, especially EGCG. However, the oral administration application is extremely restricted by the low bioavailability of EGCG. This intrigues the research interest in the potential application of EGCG as a topical treatment. This review summarizes the beneficial effects of EGCG at different skin wound healing stages. In addition to the application of EGCG in its native form, EGCG is also carried by different types of wound dressings to achieve better adhesive and infiltrative properties. EGCG can be carried/fabricated into hydrogels, nanoparticles, micro/nanofiber networks and microneedles. Abundant cell line studies and a few animal studies indicate that EGCG promotes skin wound healing based on its antioxidant, anti-inflammatory, antimicrobial, angiogenesis and antifibrotic effects and its targeting of the inflammation-related NF-κB signal pathway and fibrosis-related STAT3-signaling pathway. The possible mechanisms underlying the beneficial effects of EGCG on skin wound healing are depicted in Figure 3.

Cell line experiments are an important route to investigate the anti-inflammatory effects of bioactives, which can be roughly divided into two groups: one is the cells pretreated with bioactive products and then stimulated with an inflammatory inductor, the other is the cells firstly stimulated with an inflammatory inductor and then treated with bioactive products. Based on the simulation/treatment order, the meaning of the result could be different, which deserves careful addresses in the articles. Various models of cell studies and animal studies are used for investigating the effects of EGCG and its wound dressings on wound healing, which makes it difficult to compare the performance of EGCG-containing formulas. A standard testing method on skin wound healing is in need for evaluations of efficacy and effectiveness. Moreover, the anti-scarring results of EGCG need more evidence from clinical trials to substantiate their benefits on skin wound healing. Considering the anti-inflammatory effect of EGCG, it is postulated that the effect could be optimized if the topical product was applied shortly at an appropriate time after wounding rather than the period of re-epithelialization and a visible scar formation of wounds, which also brings up a future research direction.

## Figures and Tables

**Table 1:** The roles of EGCG and its wound dressings in the biophysiological events at different wound healing stages.

Wound Healing Stage	Role of EGCG and Its Wound Dressings	Ref.
Hemostasis	Increasing hemadsorption	[56]
Inflammation	Limiting the infiltration of neutrophils	[59]
	Inhibiting the migration and adhesion of monocytes	[60]
Proliferation	Advancing re-epithelialization	[68,75]
	Accelerating angiogenesis	[23]
	Altering collagen synthesis	[69,70,72]
	Reducing ECM formation	[70]
Remodeling	No reference about the effect of EGCG on collagen remodeling, vascular maturation and regression	

**Table 2:** The anti-inflammatory and angiogenic effects of EGCG and its wound dressings.

Compounds	Model	Treatments	Results ^1^	Ref.
EGCG	RAW 264.7	Inductor: 200 EU/mL LPS Treatment time: pretreatment with EGCG for 30 min and then stimulation with LPS for a further 24 h Culture medium: fresh medium without fetal bovine serum	Effective concentration: 20 µg/mL EGCG In culture supernatants: IL-1β↓ In cells: protein abundance of Notch signaling pathway Notch-1↓, Notch-2↓, Cleave-Notch-1↓, and Hes-1↓	[13]
	DM mouse	Wound creation: two circular, full-thickness wounds (8 mm) on the back Treatment time: daily, 14 d Groups: (1) normal group: 6 normal mice received 1% carboxymethylcellulose vehicle; (2) diabetes group: 6 DM mice were applied with 1% carboxymethylcellulose vehicle; (3) EGCG group: 6 DM mice treated with 10 mg/mL EGCG	Effective concentration: 10 µg/mL EGCG In wound tissue: compared with the diabetes group, IL-1β↓, TNFα↓, IL-6↓, the protein abundance of Notch signaling pathway Notch-1↓and Notch-2↓, macrophage accumulation↓	
EGCG	NHKs	Inductor: 250 U/mL TNFα Treatment time: coexistence treatment with EGCG and TNFα for 48 h Culture medium: fresh medium without EGF or bovine pituitary extract	Effective concentration: 1~10 µM EGCG In culture supernatants: IL-8↓	[14]
EGCG	Human body	Wound creation: a full thickness skin biopsy (5 mm diameter) on both upper inner arms Treatment time: twice daily, 1–2 weeks Groups: (1) normal group: placebo treatment; (2) EGCG group: zonal priming and direct topical treatment with EGCG	In wound tissue: the protein and mRNA abundances of VEGFA↓and CD31↓	[15]
GBE-EGCG mixture	NHKs	Inductor: 250 U/mL TNFα Treatment time: coexistence treatment with GBE (0.1 or 0.5%) and/or EGCG for 48 h Culture medium: fresh medium without EGF or bovine pituitary extract	Effective concentration for cumulative downregulating effect: 1 µM EGCG added to 0.1% and 0.5% GBE In culture supernatants: IL-8↓	[61]
EGCG-containing gold nanoparticles (AuEA)	Male BALB/c mice	Wound creation: two circular, 1 cm, full-thickness wounds on the bilateral upper back Treatment time: daily, 7 d Groups: (1) normal group: the left wound treated with vehicle; (2) AuEA group: treatment with AuEA ointment	Effective concentration: 1 mg/g EGCG + 30 mg/g α-lipoic acid (EA) + 0.07 mg/g AuNP ointment In wound tissue: CD68 protein↓, VEGF protein↑	[59]
EGCG-loaded PLGA particles	Fibroblasts	Inductor: 100 ng/mL LPS Treatment time: coexistence treatment with EGCG microparticles and LPS for 24 h Culture medium: DMEM supplemented with 10% (*v*/*v*) FBS, and 1% (*v*/*v*) penicillin/streptomycin	Effective concentration: equivalent to 400 μg/mL EGCG in microparticles In cells: expressions of TNFA↓, IL-1β↓, IL-6↓	[37]
EGCG-containing hydrogel (HA_TE)	RAW 264.7	Inductor: 100 ng/mL LPS Treatment time: the hydrogel placed on Transwell, and then stimulated with LPS for a further 24 h Culture medium: not specifically mentioned	Effective concentration: 0.1–1.0% HA_TE (*w*/*v*) In culture supernatants: TNFα↓	[20]
EGCG-chitosan hydrogel	RAW 264.7	Inductor: 100 ng/mL LPS Treatment time: stimulated with LPS for 6 h, and then treated with the collected EGCG-contained medium for 24 h Culture medium: DMEM supplemented with 10% (*v*/*v*) FBS, 1% (*w*/*v*) L-glutamine, and 1% (*v*/*v*) penicillin/streptomycin	Effective concentration: 3 mM~11 mM EGCG of the hydrogel In culture supernatants: TNFα↓	[12]
EGCG-containing EACPA hydrogel	Raw 264.7	Inductor: 1 µg/mL LPS Treatment time: stimulated with LPS for 24 h, and then treated with EACPA hydrogel extract for 24 h Culture medium: DMEM culture medium	Effective concentration: EACPA hydrogel extract with 10 mg mL^−1^ In cells: the expressions of both iNOS and IL-1β↓, the expressions of CD163, CD206 and ARG-1↑	[31]
	Diabetic C57BL/6 mice	Wound creation: full-thickness longitudinal incisions (8 mm) on the back. Treatment time: daily, 3 d Groups: (1) normal group: the wound undressed; (2) Tegaderm film group: treatment with Tegaderm film; (3) EACPA hydrogel group	Effective concentration: EACPA hydrogel with 9 mm EA complex In wound tissue: the concentrations of both IL-1β and IL-6↓, the concentrations of both IL-4 and IL-10↑	
EGCG/PLGA membrane	BALB/c nude mice	Wound creation: a full thickness skin wound (12 mm) on the upper back Treatment: implanting for 2 weeks Groups: (1) normal group: PLGA membrane implanting; (2) EGCG group: EGCG/PLGA membrane implanting	Effective concentration: EGCG/PLGA membrane containing 1 wt% EGCG In wound tissue: the immunoreactivities of Ki-67↑ and CD 31↑	[23]

^1^ Compared with control. EU: endotoxin unit; NHKs: normal human keratinocytes; DM: diabetes mellitus; RAW 264.7: a murine leukemia macrophage cell line; HUVECs: human umbilical vein endothelial cells; HASMCs: human aortic smooth muscle cells; GBE: Ginkgo biloba leaves; AuEA: gold nanoparticles + EGCG + α-lipoic acid mixture; HA_TE: EGCG-hyaluronic acids-tyramine hydrogel; EACPA hydrogel: EGCG-3-acrylamido phenyl boronic acid-acrylamide hydrogel; EGCG: epigallocatechin gallate; TP: green tea polyphenol; TPN @ H: green tea polyphenol nanospheres in PVA/alginate hydrogel; PLGA: poly (lactide-co-glycolic acid); TNFα: tumor necrosis factor α; LPS: lipopolysaccharides; EGF: epidermal growth factor; IL: interleukin; VEGF: vascular endothelial growth factor.
